# Editorial: Imaging in the diagnosis and treatment of eye diseases

**DOI:** 10.3389/fopht.2026.1899308

**Published:** 2026-06-18

**Authors:** Weihua Yang, Yalin Zheng, Shujun Wang

**Affiliations:** 1 Shenzhen Eye Hospital, Shenzhen Eye Medical Center, Southern Medical University, Shenzhen, China; 2 Department of Eye and Vision Science, Institute of Life Course and Medical Science, University of Liverpool, Liverpool, United Kingdom; 3 St Paul’s Eye Unit, Royal Liverpool University Hospital, Liverpool, United Kingdom; 4 Department of Biomedical Engineering, The Hong Kong Polytechnic University, Hong Kong, Hong Kong SAR, China

**Keywords:** ophthalmology, medical imaging, artificial intelligence, multimodal fusion, quantitative, biomarkers, diagnosis, treatment

## 1 Introduction

Ophthalmic imaging serves as the cornerstone of clinical practice and research in ophthalmology. From color fundus photography (CFP) to optical coherence tomography (OCT) and then to OCT angiography (OCTA), each technological innovation profoundly reshapes the paradigms of screening, diagnosis, and follow-up for ophthalmic diseases, including diabetic retinopathy, macular diseases, glaucoma, anterior segment abnormalities, ocular surface diseases, orbital diseases, high myopia, and even some rare conditions. In recent years, with the integration and rapid advancement of artificial intelligence (AI) into ophthalmic imaging, the landscape of ophthalmic imaging is undergoing a profound transformation from pure structural depiction toward multi-dimensional, multi-modal, functional, and quantitative integration. The era of large models brings new opportunities for the development of AI in ophthalmology (1). The construction of intelligent ophthalmology is gradually shifting from algorithm development toward a paradigm oriented toward clinical application (2), enabling the identification of early subtle lesions, individualized prediction of treatment responses, and cross-organ association analysis of systemic diseases.

Traditional static structural parameters in ophthalmic imaging are also being supplemented and deepened by more refined quantitative biomarkers. Using OCTA and advanced image processing algorithms, it is possible to obtain not only simple vessel density parameters but also indicators with pathophysiological significance for diseases, such as vessel fractal dimension, tortuosity, and choroidal vascular index (CVI) (3). These refined parameters help reveal early pathological changes in ocular diseases and provide new perspectives for quantitative assessment of disease characteristics, as well as further offering new dimensions for functional evaluation and surgical planning.

In recent years, multimodal imaging fusion strategies have greatly improved diagnostic accuracy. Compared with the traditional reliance on a single imaging modality, the multimodal combined analysis of various ophthalmic imaging techniques—such as structural information from OCT, microvascular information from OCTA, and macroscopic features from color fundus photography—has demonstrated unique advantages over any single modality in complex differential diagnoses involving the resolution of subtle structural information and morphological changes in ocular diseases (4).

The eye serves as a sensitive source of biomarkers for systemic chronic diseases, particularly cardiovascular disease, hypertension, diabetes mellitus, and peripheral neuropathy. Accumulating evidence indicates that the microvascular, neural, and biomechanical features of the eye reflect systemic pathophysiology. Ophthalmic imaging is thus breaking beyond the scope of local diagnosis and emerging as an ocular window into systemic microcirculation. Multiple studies have confirmed that ophthalmic imaging parameters, such as choroidal vascular index, retinal vessel wall thickness, and quantitative changes in corneal nerves, are significantly associated with systemic diseases including systemic sclerosis (5), thyroid-associated ophthalmopathy (6), and diabetic peripheral neuropathy (7).

Compared with its previous role as a mere auxiliary tool for diagnosis, ophthalmic imaging has been successfully integrated into various aspects of perioperative management, supporting full-process perioperative care. Ophthalmic imaging provides critical evidence for surgical safety assessment and complication prevention prior to corneal refractive surgery (8). It guides the adjustment of immunosuppressive regimens during keratoplasty (9), and predicts long-term survival of transplanted retinal pigment epithelial cells and the risk of immune rejection (10). Thus, ophthalmic imaging has become integrated into the three core stages of perioperative management—preoperative planning, intraoperative decision-making, and postoperative prediction—and has emerged as an indispensable technological pillar of ophthalmic care.

Despite the rapid development of ophthalmic imaging, major challenges remain in areas: the standardization of imaging parameters across different devices and populations, the interpretability of imaging data, and the real-world clinical integration of imaging technologies. This review systematically summarizes 43 articles on the application of imaging in the diagnosis and treatment of ophthalmic diseases, covering original research, case reports, methodological papers, and systematic reviews. It aims to comprehensively review the evolutionary process of ophthalmic imaging. This process involves a transition from structural depiction to functional integration and from local diagnosis to systemic assessment. It also aims to explore the core value and future trends of ophthalmic imaging throughout the full cycle of disease management, thereby providing a reference for clinical practice and translational research.

## 2 Refined quantitative biomarkers in ophthalmic imaging

### 2.1 Anterior segment imaging

In the field of anterior segment imaging, multiple studies have systematically demonstrated the technological evolution from static structural measurements toward dynamic functional assessment and automated grading. Abusamak et al. used anterior segment spectral-domain OCT to perform refined measurements of normal corneal stromal thickness, revealing a centrifugal distribution of corneal thickness, with the thinnest area centrally and the thickest area in the superior periphery. This finding provides important biomechanical references for safety assessment of the residual stromal bed thickness in corneal refractive surgeries such as LASIK and SMILE. Subsequently, Liu et al. extended the research perspective from static structures to dynamic changes at the cellular level. Using confocal microscopy, they found that corneal dendritic cells cause false-positive interference during automatic segmentation because they morphologically resemble nerve fibers, and that true parameters of corneal nerve fiber density, branch density, and tortuosity can only be obtained after excluding these cells. This is crucial for accurately assessing the extent of nerve damage in chronic dry eye after refractive surgery. Meanwhile, dynamic changes in corneal temperature have also been incorporated into the quantitative assessment system. Wu et al. employed high-resolution infrared thermography to quantify the dynamic changes and irregularity of corneal temperature within 10 seconds after eye opening in patients with dry eye and normal individuals, expanding the assessment dimension from static structure to functional thermodynamic parameters. Furthermore, Tang et al. developed an artificial intelligence model based on slit-lamp retroillumination images that can automatically diagnose and grade cataracts, thereby transforming traditional subjective physician-dependent grading standards into reproducible automated quantitative outputs (11). In the study by Fan et al., the porTable 3D imaging system achieved excellent reliability during standardized direct measurements of the upper eyelid and the upper eyelid crease region. In the context of high myopia, Liu et al. revealed the molecular and biomechanical alterations in the anterior segment of highly myopic eyes, correlating quantitative imaging parameters with tissue-level molecular mechanisms and expanding the application boundaries of anterior segment imaging in research on the pathological mechanisms of high myopia (12). Collectively, these studies indicate that anterior segment imaging has evolved from a single modality of static morphological depiction into a comprehensive assessment system. This system integrates biometry, cellular-level neural analysis, and dynamic thermographic monitoring, as well as automated grading, thus providing a solid imaging foundation for objective quantification, individualized diagnosis, and treatment of anterior segment diseases.

### 2.2 Retinal and choroidal imaging

In choroidal imaging, Chen et al. used ultra-widefield OCTA to perform a zonal assessment of the choroidal vascular index (CVI) in diabetic patients. They found that in patients with mild-to-moderate non-proliferative diabetic retinopathy (NPDR), the most significant decrease in CVI occurs in the far peripheral regions (the 15–18 mm temporal quadrant), and that this decrease is closely correlated with renal function parameters, suggesting that peripheral choroidal blood flow changes may serve as an early sensitive indicator of diabetic retinopathy (DR) (13). In the assessment of retinal diseases, both Song et al. and Zhou et al. have emphasized the clinical significance of hyperreflective material on OCTA. Song et al. developed deep learning models to accurately predict treatment responses at 1 day, 1 month, and 3 months after anti-VEGF therapy based on baseline OCT images, identifying the morphology and distribution of hyperreflective material as key predictors of macular edema. Zhou et al. further subclassified hyperreflective material into six subtypes. Using OCTA, they also showed that 75% to 94% of hyperreflective material precisely colocalizes with regions of intraretinal microvascular abnormalities (IRMAs), providing imaging evidence that lipoprotein extravasation is the core source of these materials. These findings have revealed quantitative imaging parameters of choroidal and retinal microvasculature, driving a paradigm shift in fundus imaging from structural description toward functional and mechanistic assessment.

## 3 Multimodal ophthalmic imaging enhances diagnostic accuracy

Traditional ophthalmic imaging diagnosis has long relied on a single imaging modality, such as color fundus photography for retinopathy screening, OCT for macular structural assessment, and OCTA for microvascular visualization. However, the information dimension of a single modality is limited, making it difficult to fully capture the complex pathological features of ocular diseases. Multimodal imaging fusion strategies, by integrating complementary information from different imaging techniques, have significantly improved diagnostic accuracy.

### 3.1 Fundus diseases

In the differential diagnosis of complex fundus diseases, multimodal ophthalmic imaging plays an irreplaceable role. Zhou et al. reported a case of a high myopia patient in whom a depression in the macular region was misdiagnosed as duplication of the optic disc, due to its morphological resemblance to the optic disc. Magnetic resonance imaging (MRI) confirmed the presence of only one optic nerve, OCT revealed a steep scleral depression without lamina cribrosa structures, and fundus fluorescein angiography (FFA) demonstrated blood supply originating from the short posterior ciliary arteries. Through combined multimodal imaging analysis, the final diagnosis of myopic macular pit was established, avoiding misdiagnosis and treatment bias. This study demonstrates that multimodal imaging fusion strategies, by integrating complementary information from different imaging techniques, effectively overcome the inherent limitations of any single modality and exhibit strong potential in complex differential diagnostic settings.

### 3.2 Rare diseases

Zhou et al. reported a case in which two independent primary orbital melanomas developed in the same orbit nine years after ocular content evisceration for congenital corneal staphyloma. Using MRI, positron emission tomography-computed tomography (PET-CT), and pathological immunohistochemistry, the possibility of metastasis was excluded. Tracing back to the patient’s widespread blue nevi throughout the body, the authors proposed that blue nevus syndrome might be a risk factor for primary orbital melanoma. Xi et al. reported a patient presenting with monocular central retinal artery occlusion (CRAO) as the initial manifestation, in whom conventional antithrombotic therapy was ineffective. Further imaging examinations combined with serological findings revealed positivity for c-ANCA, leading to a final diagnosis of granulomatosis with polyangiitis (GPA). This case underscores the importance of combining ophthalmic imaging with systemic evaluation when encountering atypical CRAO.

## 4 Application of ophthalmic imaging in systemic diseases

The eye is often described as a window into systemic diseases, and many studies have focused on extracting ophthalmic imaging biomarkers associated with cardiovascular and metabolic conditions. A substantial body of research in ophthalmic imaging has been dedicated to extracting quantifiable biomarkers from images and linking them to the risk and prognosis of systemic diseases.

### 4.1 Cardiovascular and metabolic diseases

Multiple studies have confirmed that retinal microcirculation parameters are sensitive indicators of systemic cardiovascular health. Luo et al. used ultra-widefield SS-OCTA to examine patients with severe carotid artery stenosis undergoing carotid revascularization. The results showed that retinal perfusion in both the macular and peripheral regions of the ipsilateral eye significantly improved, along with a marked decrease in the area and perimeter of the foveal avascular zone, further supporting the clinical value of retinal microcirculation parameters as imaging biomarkers of vascular status in the carotid artery system. This reveals the potential of ocular parameters as imaging biomarkers for systemic neuropathy. Cao et al. employed OCTA to investigate and observed that retinal arteriolar density in patients with intracranial artery stenosis was significantly lower than that in patients with extracranial artery stenosis, and that hypertension was significantly correlated with reduced retinal arteriolar density (14). Raciborska et al. evaluated the symmetry of choroidal vascular index (CVI) between the two eyes in patients with systemic sclerosis. They found that although patients with systemic sclerosis exhibit significant choroidal microvascular damage, the degree of damage is highly symmetric between the two eyes. This finding suggests that monocular data can be used for assessment in clinical studies and provides evidence for understanding the symmetry of systemic vasculopathy in systemic sclerosis.

### 4.2 Inflammation, immunity, and tumors

In certain systemic diseases arising from immune dysregulation or malignancy, the eye is often the first organ affected. Peng et al. documented an elderly patient with unilateral choroidal detachment and bilateral serous retinal detachment as the presenting features, and the patient was ultimately diagnosed as Vogt-Koyanagi-Harada syndrome (15). This case illustrates that in elderly individuals, Vogt-Koyanagi-Harada syndrome may present as atypical unilateral choroidal detachment, and hypoperfusion areas of the choroid revealed by indocyanine green angiography, as a form of multimodal imaging, serve as the key to differential diagnosis. Li et al. described a case of retinal metastasis from lung adenocarcinoma. The patient exhibited yellow-white fundus lesions accompanied by hemorrhage, with OCT and fluorescein angiography features distinctly different from those of infectious retinitis. This report provides important diagnostic considerations for vision loss in patients with end-stage cancer.

## 5 Ophthalmic imaging facilitates perioperative management, treatment response prediction, and long-term follow-up

### 5.1 Perioperative planning and complication prediction

In the field of refractive surgery, Zhang et al. compared the repeatability of corneal curvature measurements obtained by five devices in patients with and without dry eye. They found that dry eye status significantly increases measurement variability. However, the ZEISS IOL Master 700, based on SS-OCT principles, and the NIDEK OPD-Scan III, a wavefront-based aberrometer, displayed superior stability and reliability, providing evidence-based guidance for selecting appropriate measurement devices in dry eye patients prior to refractive surgery. In the field of keratoplasty, Zhang et al. employed reflectance-mode scanning laser ophthalmoscopy (RM-SLO) to detect soft drusen and observed that its detection rate was significantly higher than that of OCT and CFP, with the added ability to reveal pseudo-three-dimensional morphology. Ji et al. developed an automatic grading system for pterygium based on semantic segmentation. By precisely segmenting the cornea, pupil, and pterygium regions and incorporating a curve-fitting algorithm to quantify the depth of infiltration, the system achieved automated grading with high consistency with expert assessments, offering an objective tool for preoperative evaluation and remote screening of pterygium. With two-year follow-up data, Gong et al. constructed a predictive model that enables individualized assessment of the risk of postoperative cataract development, thereby providing an important reference for perioperative management following vitreoretinal surgery (16).

### 5.2 Imaging prognostic markers for drug injection therapy

For patients with macular edema receiving anti-VEGF therapy, features on baseline OCT images currently represent the most extensively investigated prognostic markers. Beyond the hyperreflective material discussed earlier, Di et al. combined a novel near-infrared fluorescent nanoprobe for tracking transplanted retinal pigment epithelial cells in a mouse model. This nanoprobe offers favorable biocompatibility and photostability. Its signal intensity and distribution can reflect the viability of transplanted cells as well as the extent of immune rejection, thereby furnishing a powerful imaging assessment platform for the clinical translation of regenerative medicine.

### 5.3 Dynamic monitoring of treatment response

In the long-term follow-up of glaucoma, Lv et al. designed an automated system for quantifying cup-to-disc ratio from color fundus photographs, offering an efficient and objective structural assessment tool for community-based screening of chronic glaucoma (17). Wu et al. performed longitudinal monitoring of vessel density in the macular and optic disc regions of healthy eyes, glaucoma suspect eyes, and eyes with primary open-angle glaucoma (POAG) using OCTA. Their results showed that the annual reduction rates of whole-image vessel density (wiVD) in the macular region across the three groups ranged from 0.72% to 0.92%, and those of whole-image capillary density (wiCD) in the optic disc region ranged from 0.28% to 0.66%, both significantly faster than zero (p < 0.05) (18). Lin et al. applied *in vivo* confocal microscopy (IVCM) to monitor rejection after penetrating keratoplasty. They found that the characteristic immune activation response of the endothelium serves as an early sign of rejection, and that when rejection occurs, the most prominent subjective symptom reported by patients is decreased visual acuity. Collectively, these studies indicate that imaging parameters have become core metrics for evaluating treatment efficacy and monitoring disease outcomes.

## 6 Current challenges and future perspectives of ophthalmic imaging in the diagnosis and treatment of ocular diseases

Despite the considerable progress achieved in ophthalmic imaging, major challenges still persist, including the lack of standardized imaging parameters across different devices and populations, the interpretability of imaging data, and the real-world clinical integration of imaging technologies. To enable high-quality application of ophthalmic imaging in clinical practice and research, efforts should extend beyond merely pursuing improved accuracy of algorithms on ideal datasets. Instead, under the complex conditions of real-world clinical settings, it is essential to establish an imaging application system that is spatiotemporally robust, reproducible, trustworthy, and practically implementable. This will better advance ophthalmic care toward the goal of precision medicine.

### 6.1 Multimodal and spatiotemporal radiomics

At present, the evaluation of ophthalmic disease diagnosis and treatment remains predominantly reliant on static structural assessments. This approach not only struggles to capture the spatiotemporal heterogeneity of disease evolution but also fails to enable true dynamic comparisons of longitudinal follow-up data from the same patient. Future advancements in ophthalmic imaging will build upon conventional radiomics by incorporating both temporal and spatial dimensions. Through high-throughput feature extraction and dynamic modeling of images acquired from the same patient and the same lesion at varying time points and spatial locations, the evaluative framework of ophthalmic imaging will be elevated from static snapshots taken at a single time point to dynamic imaging that spans the entire disease continuum. This transformation will furnish a completely new methodological foundation for deciphering the spatiotemporal evolution patterns underlying the onset, progression, and therapeutic response of ocular diseases.

### 6.2 Standardization and reproducibility

As noted earlier, different measurement devices lack consistency and accuracy in acquiring corneal curvature data from patients with dry eye disease (19). Factors such as race, age, refractive status, and ocular phenotype exert considerable influence on image quality and parameter quantification. Furthermore, variations in imaging principles, resolution, and signal-to-noise ratio across different manufacturers and device models further complicate direct comparability of measurements obtained from the same patient using different instruments. Future efforts should focus on establishing cross-device calibration procedures and standardized image acquisition protocols, as well as developing robust analytical algorithms capable of accommodating such heterogeneity, thus enhancing the standardization of ophthalmic imaging data collection and analysis.

### 6.3 Interpretability and trustworthiness

With the rapid advancement of artificial intelligence, deep learning models have achieved remarkable accuracy in automated segmentation, lesion detection, and disease classification within ophthalmic imaging. Nevertheless, the black-box nature of AI continues to pose considerable challenges to evidence-based practice and clinical decision support. Zhou et al. subclassified hyperreflective material on OCTA images of diabetic retinopathy into six subtypes and elucidated its spatial association with vascular abnormalities and systemic lipid dysregulation, demonstrating that the significance of such research extends beyond technical classification. Rather, it provides a potential pathophysiological explanation, specifically lipoprotein extravasation, for the observed imaging phenomena. Some researchers have also noted that artificial intelligence should serve as an empowering assistant in ophthalmology rather than a black-box system that replaces physician decision-making (20). Therefore, future research in ophthalmic imaging should place greater emphasis on integrating algorithmic outputs with causal reasoning of pathological mechanisms, so as to enhance image interpretability and build intelligent analysis systems that clinicians can trust.

### 6.4 Precision and personalization

Currently, we primarily depend on morphological parameters derived from ophthalmic imaging to diagnose the current status of ocular diseases. Future work should be directed toward developing imaging phenotypes with predictive capabilities, focusing on forecasting disease trajectories and treatment responses using baseline imaging data. Di et al. analyzed fractal dimension of the choroidal vasculature, textural features of the retinal nerve fiber layer, and cellular morphology under corneal confocal microscopy. By integrating these parameters with large-scale clinical data, they constructed a model capable of predicting disease progression risk and treatment response probability. Enhanced predictive power will transform ophthalmic imaging from a diagnostic tool into a decision-support partner for clinicians, thus further accelerating the progress of precision medicine.

### 6.5 From algorithmic maturity to clinical ecological maturity

The transition of imaging technologies from algorithmic maturity in the laboratory to clinical ecological maturity still faces numerous obstacles. Cost-effectiveness analyses of emerging technologies such as portable three-dimensional imaging devices or smartphone-based slit-lamp adapters (21, 22), coverage by medical insurance reimbursement systems, training and acceptance among ophthalmologists and technicians, as well as the accompanying pressures of data storage and privacy protection, all represent practical factors restricting their widespread adoption. Consequently, the future evolution of ophthalmic imaging should extend beyond the pursuit of higher resolution and more sophisticated algorithms. It will also require the construction of a collaborative innovation ecosystem that includes device developers, clinicians, health economists, and policy makers. Such an ecosystem will collectively facilitate the clinical translation of advanced imaging technologies and ultimately benefit patient care. Recently, researchers introduced the Reti-Pioneer, a multi-task retinal imaging framework based on color fundus photographs. This framework is capable of detecting six common metabolic disorders, including type 2 diabetes, hypertension, hyperlipidemia, gout, osteoporosis, and thyroid disease, offering a low-cost pathway for clinical translation (23).

## 7 Conclusion

Ophthalmic imaging is rapidly reshaping the landscape of both research and clinical practice in eye diseases. From the centrifugal distribution of corneal stromal thickness, to zonal variations in the choroidal vascular index, and to subtype-specific spatial localization of hyperreflective material, an increasing amount of biological meaning and clinical decision-making weight is being assigned to ophthalmic imaging parameters. Despite the continuous breakthroughs in ophthalmic imaging technologies, major challenges still persist, including the lack of standardization of imaging parameters across different devices and populations, the interpretability of imaging data, and the real-world clinical integration of imaging techniques, all of which are complexly intertwined. Establishing an ophthalmic imaging framework characterized by standardization, interpretability, and precision across heterogeneity in devices and populations holds promise for addressing these issues. In the future, ophthalmic imaging will evolve from refined quantitative biomarkers to multimodal fusion diagnostics, from an ocular window into systemic diseases to perioperative decision support, and from static cross-sectional snapshots to dynamic stereoscopic sequences enabled by higher-dimensional spatiotemporal omics. This evolution will enable ophthalmic imaging not only to capture the microenvironmental heterogeneity of disease progression but also to serve as a decision-making partner for clinicians throughout the process of diagnosis and treatment. It will reshape the full-cycle management paradigm for ophthalmic diseases and consequently propel ophthalmic healthcare toward greater advancements.
